# Steroidogenic Acute Regulatory Protein (StAR): Evidence of Gonadotropin-Induced Steroidogenesis in Alzheimer Disease

**DOI:** 10.1186/1750-1326-1-14

**Published:** 2006-10-03

**Authors:** Kate M Webber, Douglas M Stocco, Gemma Casadesus, Richard L Bowen, Craig S Atwood, Laura A Previll, Peggy LR Harris, Xiongwei Zhu, George Perry, Mark A Smith

**Affiliations:** 1 Department of Pathology, Case Western Reserve University, Cleveland, Ohio, USA; 2 Department of Cell Biology & Biochemistry, Texas Tech University Health Sciences Center, Lubbock, Texas, USA; 3 Voyager Pharmaceutical Corporation, Raleigh, North Carolina, USA; 4 School of Medicine, University of Wisconsin and William S. Middleton Memorial Veterans Administration, Madison, Wisconsin, USA.; 5 College of Sciences, University of Texas at San Antonio, San Antonio, Texas, USA; 6 Raleigh, North Carolina, USA

## Abstract

**Background:**

Alzheimer disease (AD) is clinically characterized by progressive memory loss, impairments in behavior, language and visual-spatial skills and ultimately, death. Epidemiological data reporting the predisposition of women to AD has led to a number of lines of evidence suggesting that age-related changes in hormones of the hypothalamic-pituitary-gonadal (HPG) axis following reproductive senescence, may contribute to the etiology of AD. Recent studies from our group and others have reported not only increases in circulating gonadotropins, namely luteinizing hormone (LH) in individuals with AD compared with control individuals, but also significant elevations of LH in vulnerable neuronal populations in individuals with AD compared to control cases as well as the highest density of gonadotropin receptors in the brain are found within the hippocampus, a region devastated in AD. However, while LH is higher in AD patients, the downstream consequences of this are incompletely understood. To begin to examine this issue, here, we examined the expression levels of steroidogenic acute regulatory (StAR) protein, which regulates the first key event in steroidogenesis, namely, the transport of cholesterol into the mitochondria, and is regulated by LH through the cyclic AMP second messenger pathway, in AD and control brain tissue.

**Results:**

Our data revealed that StAR protein was markedly increased in both the cytoplasm of hippocampal pyramidal neurons as well as in the cytoplasm of other non-neuronal cell types from AD brains when compared with age-matched controls. Importantly, and suggestive of a direct mechanistic link, StAR protein expression in AD brains colocalized with LH receptor expression.

**Conclusion:**

Therefore, our findings suggest that LH is not only able to bind to its receptor and induce potentially pathogenic signaling in AD, but also that steroidogenic pathways regulated by LH may play a role in AD.

## Background

Alzheimer disease (AD), the leading cause of senile dementia, is characterized by selective neuronal degeneration affecting the hippocampus and to a lesser extent other cortical brain regions resulting in progressive memory loss, impairments in behavior, language and visual-spatial skills and ultimately, death [1]. The etiologic events that lead to the neuronal loss and dysfunction seen in AD are not well understood; however, there are many lines of evidence that suggest the sex steroids, estrogen and testosterone, may play an important role in the pathogenesis of AD. Epidemiological studies exploring gender differences in AD have resulted in conflicting data, yet most studies support the higher prevalence [2-5] and incidence [6] of AD in women. It is our hypothesis that hormones of the hypothalamic-pituitary-gonadal (HPG) axis, especially age-related increases in gonadotropins, play a key role in AD pathogenesis [7].

There is mounting evidence that supports a role for gonadotropins, and particularly luteinizing hormone (LH), in AD pathogenesis beginning with the finding of a two-fold increase in circulating gonadotropins in individuals with AD compared with age-matched control individuals [8,9]. Significant elevations of LH were also found in vulnerable neuronal populations in individuals with AD compared to aged control cases [10]. Furthermore, LH has been shown to alter amyloid β protein precursor (AβPP) processing toward the amyloidogenic pathway [11], as well as lead to cognitive decline in LHβ-transgenic mice [12] that exhibit elevated LH levels and increased estradiol and testosterone levels when compared to non-transgenic littermates [13]. Finally, decreases in serum LH levels by leuprolide acetate (a potent gonadotropin-lowering agent) administration have been associated with decreases in amyloid plaque burden and subsequent increases in cognition in AβPP transgenic mice [12]. While evidence for the role of LH in the pathogenesis of AD is increasing, the mechanisms by which LH contributes to neuronal dysfunction or death within the course of the disease remains to be elucidated. In fact, although it has been well documented that neurons in various regions of the central nervous system synthesize sex steroids (for review see [14]) that are believed to be important for complex neuronal functions (for review see [15]), little is currently known about the etiology or effects of gonadotropins, which regulate the synthesis and secretion of sex steroids (for review see [16]), on the hippocampus.

In contrast to the consequences of LH binding to its receptor in the hippocampus, the signaling cascade initiated upon LH binding to its receptor on gonadal tissues is well documented (for review see [17]). Upon binding to its receptor, LH initiates the activation of membrane-associated adenylyl cyclase, causing an elevation of intracellular cAMP [18], which serves as a second messenger for the upregulation of the steroidogenic acute regulatory protein (StAR) primarily through the protein kinase A (PKA) pathway [17,19-21]. StAR transports cholesterol to the inner mitochondrial membrane [22] where it is converted to pregnenolone by the cytochrome P-450 enzyme complex, which represents the rate limiting step in steroidogenesis. The presence of steroidogenic enzymes within the brain, such as aromatase, which is the enzyme involved in the last step in the synthesis of estrogens as it converts testosterone to estrogen, was described over thirty years ago [23], although it has only recently been widely accepted that aromatase is expressed in both neurons and glial cells in the hippocampus as shown at the mRNA level [24] and protein level [25-27]. Like aromatase, while the presence of StAR in the brain has been documented for sometime [28], it has only recently been shown to be expressed specifically in pyramidal neurons, granule cells of the dentate gyrus, interneurons and glial cells [29].

In this study, we found an increase in StAR in AD hippocampal neurons as well as other non-neuronal cells compared to aged matched controls by immunocytochemical methods. Furthermore, we found that StAR colocalizes to neurons with LH receptor, which is expressed throughout the hippocampus [30] and is known to initiate a signaling cascade leading to increased StAR expression when bound by its ligand [17,19-21]. This finding is suggestive of a direct mechanistic link by providing further support that LH is indeed able to bind to its receptor and induce potentially pathogenic signaling in AD, and also that steroidogenic pathways regulated by LH may play a role in AD.

## Results

**Figure 1:**
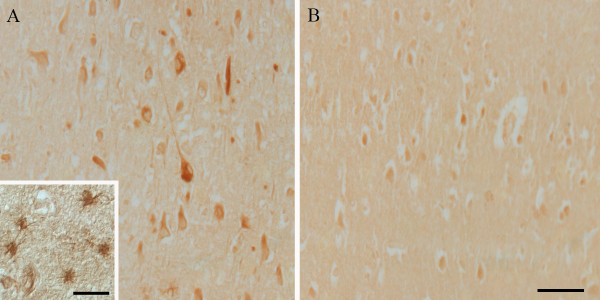
The immunolocalization of StAR. Immunocytochemical localization of StAR in AD-affected individuals is increased in pyramidal hippocampal neurons as well as in astrocytes (Panel A) in comparison to age-matched control brains (Panel B). Scale bar for panels A and B = 50 μm. Scale bar within the inset in Panel A = 25 μm.

**Figure 2:**
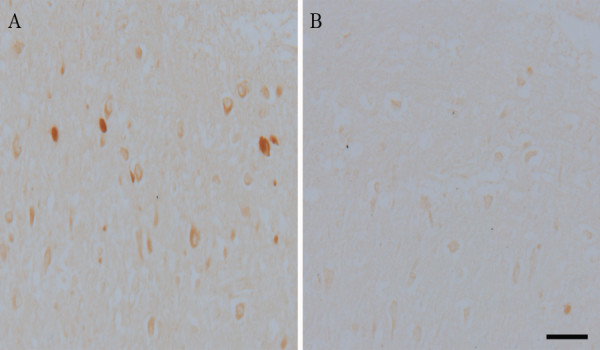
Absorption verifies the specificity of antibody binding. Staining in the hippocampus with StAR in AD (Panel A) is decreased by absorption with the immunizing peptide (Panel B). Scale bar = 50 μm.

StAR protein was markedly increased in both the cytoplasm of hippocampal pyramidal neurons as well as in the cytoplasm of other cell types, such as astrocytes, from sixteen AD brains when compared with twelve age-matched controls (Fig. 1). Furthermore, while StAR was predominantly found in the cytoplasm, it also localized to neurofibrillary tangles, neuropil threads and dystrophic neurites (Fig. 1). No significant StAR was noted in the cerebral vasculature. To confirm the specificity of StAR immunocytochemistry, several control experiments were performed in parallel. Absorption of StAR antibody with excess immunizing peptide was shown to appreciably decrease immunostaining (Fig. 2), and omission of primary antibody resulted in no apparent staining.

**Figure 3:**
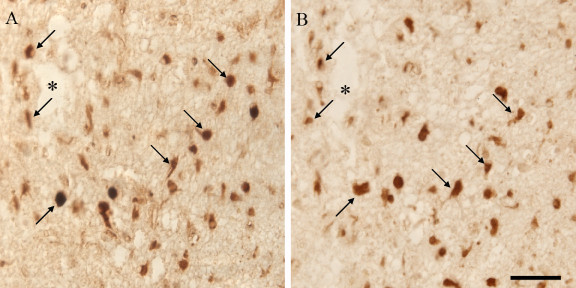
Immunocytochemical analysis of the distribution of StAR and hLH/CG receptor. Adjacent serial hippocampal sections of an AD patient show overlap between StAR immunolocalization (Panel A) and hLH/CG receptor immunolocalization (Panel B). Arrows point to neurons included in both hippocampal sections that show positive immunoreactivity for StAR and LH receptor. Asterisk denotes landmark vessel. Scale bar = 50 μm.

Colocalization experiments using adjacent AD hippocampal serial sections were performed in order to determine if cells expressing StAR protein also expressed the human LH receptor. StAR colocalized with LH receptor in both pyramidal neurons (Fig. 3) as well as in other cells types (data not shown) suggesting that LH, which is known to be increased in AD [10], is in fact able to bind to its receptor and initiate canonical signaling cascades in the hippocampus despite the non-gonadal nature of the tissue. It should be noted that follicle-stimulating hormone (FSH) and adrenocorticotropic hormone (ACTH) are also known to increase StAR expression ([31] and [32], respectively); however, we did not include the receptors for these hormones in this colocalization study as the FSH receptor has not been reported in the brain and ACTH is decreased in AD [33,34].

## Discussion

In this study, we demonstrate increased levels of StAR protein in vulnerable neurons as well as in other cell types in individuals with AD when compared to the same cellular populations in normal, aged-matched individuals (Fig. 1). The expression of StAR in different hippocampal cell types in this study mimics recent findings in which StAR localized to not only pyramidal neurons, but also to granule cells of the dentate gyrus, interneurons and glial cells in marmosets and rats [29]. We also demonstrate that StAR colocalizes to neurons expressing LH receptor as demonstrated on adjacent serial sections of AD hippocampal tissue (Fig. 2). Considering that LH is significantly elevated in vulnerable neuronal populations in individuals with AD [10] and that the highest LH receptor expression in the brain is in the hippocampus [30], the colocalization of StAR with LH receptor is highly suggestive of a mechanistic link. Data from this study not only suggests that LH is able to bind to its receptor and induce signaling cascades in non-gonadal tissue, but also that the steroidogenic consequences of increased LH binding may play a role in AD pathogenesis. With this in mind, it is important to note that the long held notion of the pathogenic effects of decreased sex steroid levels, namely estrogen, on the brain after reproductive senescence is not consistently reflected in studies measuring sex steroid levels in AD compared to age-matched controls. For example, only two of nine recent observational studies comparing estrogen levels in women with AD with controls reported lower estrogen levels in AD [35,36], with two studies reporting increased estrogen levels in AD [37,38] and five reporting no significant differences between AD and controls [39-43]. The variability in the results of these studies is thought to be caused in part by the sensitivity of the assay used, as studies that used less sensitive assays reported higher total estrogen levels [38] resulting in an overemphasis of the impact of low estrogen levels on the study. Therefore, potentially elevated sex steroid levels resulting from increased StAR expression in AD demonstrated in this study is not necessarily contradictory to epidemiological studies regarding sex steroid levels AD.

While a consensus on sex steroid levels in AD remains elusive, studies involving the role of gonadotropins, and in particular the role LH, in AD pathogenesis support increases in gonadotropin-induced steroidogenesis in the hippocampus. Epidemiological studies concerning gender differences in AD support the higher prevalence [2-5] and incidence [6] of AD in women. This has led to a number of lines of evidence suggesting that age-related changes in hormones of the HPG axis following reproductive senescence, may contribute to the etiology of AD. Due to the ineffectiveness of hormone replacement therapy in the treatment of AD [44], it is our hypothesis that age-related changes to gonadotropin levels, namely LH, contribute to AD pathogenesis. The initial finding of a two-fold increase in circulating gonadotropins in individuals with AD [8,9] was further supported when significant elevations of LH were also found in vulnerable neuronal populations in individuals with AD compared to aged control cases [10]. While this increase in serum LH in AD would not be expected to result in increased sex steroid production in the gonads due to the loss of function after reproductive senescence, increased neuronal LH in AD would likely induce steroidogenesis in functioning neurons. In support of this notion, reported decreases in steroidogenic enzyme expression, including StAR, in the post-menopausal ovary when compared to the premenopausal ovary [45] are in stark contrast to the increased levels of StAR reported in this study.

LH has also been linked mechanistically to AD as evidenced by the effect of LH on AβPP processing toward the amyloidogenic pathway as evidenced by increased secretion and insolubility of Aβ, decreased AβPP-α secretion, and increased AβPP-C99 levels [11]. This same study also reported a 3.5-fold and a 1.5-fold reduction in total brain Aβ1–42 and Aβ1–40 concentrations, respectively, in C57Bl/6J mice treated with leuprolide acetate, a potent gonadotropin-releasing hormone agonist shown to effectively lower LH serum levels [11]. Decreases in serum LH levels by leuprolide acetate administration have also been associated with decreases in amyloid plaque burden and subsequent increases in cognition in AβPP transgenic mice [12]. Even more importantly, LHβ-transgenic mice that exhibit elevated LH levels well as increased estradiol and testosterone levels [13] showed cognitive deficits specifically related to learning and memory when compared to non-transgenic littermates [12].

## Conclusion

**Figure 4:**
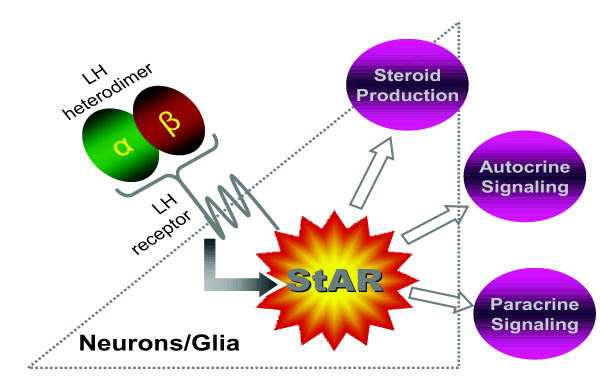
Schematic of LH action in AD. Upon binding to its receptor on neurons and/or glial cells within the brain, LH potentially induces signaling cascades that result in increased StAR expression and possibly subsequent increases in steroidogenesis via autocrine or paracrine signaling mechanisms.

In conclusion, we have shown that StAR is upregulated in both hippocampal pyramidal neurons and non-neuronal cell types in AD, and that StAR expression colocalizes with LH receptor expression in this same cellular population. This finding is suggestive of a direct mechanistic link by suggesting that LH is able to bind to its receptor and induce potentially pathogenic signaling despite the non-gonadal nature of the hippocampus, and also that steroidogenic pathways regulated by LH may play a role in AD (Fig. 4). This study provides the first connection between gonadotropins and sex steroids in the brain and offers insight into the mechanism of LH-induced pathogenesis in AD.

## Materials and methods

### Tissue

Hippocampal or cortical tissue samples were obtained post mortem from patients (n = 16, ages 67–96 years, mean = 84.7 years) with clinically and histopathologically confirmed AD, as well as from aged-matched controls (n = 12, ages 66–86 years, mean = 76.4 years) with similar post mortem intervals (AD: 5.5–25 h, mean = 9.6 h; controls: 6–27 h, mean = 13.4 h). All cases were categorized based on clinical and pathological criteria established by CERAD and NIA consensus panel [46]. From the clinical reports available to us, we found no obvious differences in agonal status or other potential confounders between the groups. Tissue was fixed in methacarn (methanol: chloroform: acetic acid; 6: 3: 1 v/v/v) at 4°C overnight. Following fixation, tissue was dehydrated through ascending ethanol, embedded in paraffin, and 6-μm sections were placed on silane-coated slides (Sigma, St. Louis, MO, USA).

### Immunocytochemistry

Tissue sections were deparaffinized in xylene, hydrated through descending ethanol, and endogenous peroxidase activity was quenched by thirty minute incubation in 3% hydrogen peroxide in methanol. Non-specific binding sites were blocked with thirty minute incubation in 10% normal goat serum. Tissue sections were immunostained with a rabbit polyclonal antibody to StAR (1:100) [47] and/or a rabbit polyclonal antibody to the human luteinizing hormone receptor (1:50) (GeneTex, Inc., TX, USA) followed by the peroxidase-antiperoxidase method with 3-3'-diaminobenzidine as co-substrate as previously described. Sections were also immunostained with a monoclonal mouse antibody, AT8 (1:1000), which recognizes phosphorylated tau (Ser202/Thr205) (Pierce, Rockford, IL) to identify the location of neuronal pathological structures. Control experiments included omission of primary antisera. Absorption experiments were performed to verify the specificity of antibody binding. StAR antigen [47] (20 mg), was linked to Aminolink Plus Coupling Gel (Pierce, Rockford, IL) following manufacturers directions, and incubated together with diluted antibody for 16 hours at 4°C on a rotator. After centrifugation, the "adsorbed" antibody solution was used for immunocytochemistry. For this experiment, the "unadsorbed" antibody was prepared by incubating diluted StAR antibody with the coupling gel only.

## Declaration of Competing interests

Drs. Atwood, Smith, and Perry serve, or have served, as consultants to, and own stock options in, Voyager Pharmaceutical Corporation.

## Authors' contributions

Data acquisition: Webber, Casadesus, Previll, Harris

Data interpretation: Zhu, Perry, Smith

Conception and Design: Smith, Stocco, Bowen, Atwood

Writing: Webber, Stocco, Smith
